# Supplementary material for: The Role of Globularia alypum Explored Ex Vivo In Vitro on Human Colon Biopsies from Ulcerative Colitis Patients
Source: Nutrients. 2023 Mar 17;15(6):1457. doi: 10.3390/nu15061457 (PMC10056518; doi:10.3390/nu15061457)
Supplement: Supplementary file 1 [file nutrients-15-01457-s001.zip › nutrients-2196993-supplementary.pdf]

Table S1. Immunostaining cell count for COX-2. Results are shown as mean±SD, p=t-test for unpaired data.

COX-2	CONTROLS	UC	p
Medium	24,6 ± 15,4	49,1±10,25	0,0001
+ LPS	39,5 ± 19,7	68,8 ±14,03	0,0001
+LPS +GAAE 50	37,5 ± 19,8	60,0 ± 21,9	0,0001
+LPS GAAE 100	30,0 ± 18,1	55,4 ± 13,6	0,0001
+ GAAE	20,8 ± 16,2	54,9 ±15,2	0,0001

Table S2 Immunostaining evaluation for ICAM-1. Results are shown as intensity of staining from 0 (absent) to 1 (mild), 2 (medium) , and 3 (high), p= ANOVA.

ICAM-1	CONTROLS	UC	p
Medium	1,58± 0,9	1,72±0,6	0,509
+ LPS	2,66 ± 0,9	2,88 ±0,5	0,292
+LPS +GAAE 50	1,50 ± 0,7	1,73 ± 0,7	0,017
+LPS GAAE 100	1,58 ± 0,6	2,16± 0,74	0,0029
+ GAAE	0,88 ± 0,6	2,04 ±0,7	0,0001

Table S3 Immunostaining evaluation for NF-KB. Results are shown as intensity of staining from 0 (absent, to 1 (mild) to 2 (medium) and 3 (high), p= ANOVA.

NFKB	CONTROLS	UC	p
Medium	1,38 ± 0,5	1,81± 0,5	0,007
+ LPS	3,00 ± 0,1	2,75 ±0,5	0,132
+LPS +GAAE 50	2,75 ± 19,8	3,0 ± 0,9	0,062
+LPS GAAE 100	2,00 ± 0,0	2,06 ± 0,7	0,800
+ GAAE	1,00 ± o	1,75 ±0,8	0,047

Table S4 Immunostaining evaluation for p38 MAPK. Results are shown as intensity of staining from 0 (absent) to 1 (mild), 2 (medium), and 3 (high); p= ANOVA.

p38 MAPK	CONTROLS	UC	p
Medium	2,0± 0,5	1,80± 0,2	0,330
+ LPS	2,75 ± 0,4	2,8 ±0,4	0,794
+LPS +GAAE 50	1,75 ± 0,7	3,0± 0,2	0,140
+LPS GAAE 100	1,86 ± 0,4	2,33 ± 0,7	0,120
+ GAAE	1,50 ± 0,5	1,93 ±0,4	0,0709

Table S5. Evaluation of immunostaining of the different inflammatory biomarkers investigated compared to each medium, significance at p>0,05 (ANOVA)

Controls	COX -2<br>p	ICAM-1<br>p	NFKB<br>p	p38 MAKP<br>p
+ LPS	0,390	**0,001**	**0,001**	0,745
+LPS +GAAE 50	0,315	0,087	0,087	0,120
+LPS GAAE 100	0,316	0,721	0,721	0,721
+ GAAE	0,098	0,334	0,334	0,374
UC	COX-2<br>p	ICAM-1<br>p	NFKB<br>p	p38 MAKP<br>p
+ LPS	**0,047**	**0,047**	**0,005**	**0,022**
+LPS +GAAE 50	**0,051**	0,667	0,511	**0,005**
+LPS GAAE 100	0,414	0,150	0,150	**0,003**
+ GAAE	0,276	**0,024**	**0,049**	0,275
